# Local Sleep Oscillations: Implications for Memory Consolidation

**DOI:** 10.3389/fnins.2019.00813

**Published:** 2019-08-20

**Authors:** Maya Geva-Sagiv, Yuval Nir

**Affiliations:** ^1^Department of Neurosurgery, University of California, Los Angeles, Los Angeles, CA, United States; ^2^Sagol School of Neuroscience, Tel Aviv University, Tel Aviv, Israel; ^3^Department of Physiology and Pharmacology, Sackler School of Medicine, Tel Aviv University, Tel Aviv, Israel; ^4^Functional Neurophysiology and Sleep Research Lab, Tel-Aviv Sourasky Medical Center, Tel Aviv, Israel

**Keywords:** sleep, memory, spindles, ripples, coherence, regional

## Introduction

Accumulating evidence suggests that sleep is important for plasticity and memory consolidation (Maquet, 2001; Walker and Stickgold, 2004; Datta and Maclean, 2007; Diekelmann and Born, 2010; Tononi and Cirelli, 2014; Dudai et al., 2015)—the transformation of new labile memories encoded in wakefulness into stable representations that integrate into long-term memory networks. A central model accounting for memory consolidation during sleep is that of coupling between hippocampal (HC) and neocortical networks (Buzsáki, 1996). According to this two-stage model of memory formation [also termed the hippocampal—neocortical dialogue model (Buzsáki, 1989)], the dominant direction of information flow across the brain differs between wake and sleep periods. During wakefulness, acquisition of sensory information mainly drives signal propagation from cortex to hippocampus (HC) (Buzsáki, 1998; Mormann et al., 2008). By contrast, during subsequent non-rapid eye movement (NREM) sleep, this model suggests a central role for information flow from HC to cortex especially around sharp-wave ripples (SWRs) events (Buzsáki, 1998). Accordingly, slow waves that originate in the neocortex repeatedly reactivate the newly encoded HC information when SWRs occur, driving subsequent activity in select cortical circuits (Siapas and Wilson, 1998). However, it is clear that information flow is not strictly unidirectional (Wagner et al., 2010) and may involve complex loops (Rothschild et al., 2017). HC reactivation tends to co-occur with sleep spindles that optimize plasticity (Seibt et al., 2017), resulting in long-term modification of synaptic efficacy. Thus, hippocampal–neocortical coupling requires interregional cross-frequency coordination between sleep oscillations, including slow waves and sleep spindles in thalamo-cortical circuits as well as HC ripples.

The underlying prevalent assumption is that sleep oscillations (slow waves in particular) are global events that co-occur nearly simultaneously across different brain regions. But in fact, they have been described as traveling waves propagating from anterior-to-posterior cortex (Massimini et al., 2004), and they typically occur out of phase across different cortical sites (Nir et al., 2011; Vyazovskiy et al., 2011; Malerba et al., 2019). How can we reconcile models requiring co-occurrence of sleep oscillations with accumulating evidence of non-uniform timing of oscillations across the brain? In this article, we first review the current data that sheds light on this question, and highlight recent studies that link *regional* coupling of sleep oscillations with consolidation of specific memories. Then, we highlight the gap between sleep and memory theory and experimental evidence. Based on studies that monitor and manipulate specific cortical circuits, we propose that coupling can occur between sleep oscillations in general, and between HC and cortex specifically, but that such coupling likely involves different brain regions at each point in time, contributing to memory consolidation in select circuits.

## Slow Waves, Spindles, and Their Regional Modulation Following Learning

Slow waves and sleep spindles constitute electroencephalographic (EEG) hallmarks of NREM sleep (Gibbs and Gibbs, 1950; Steriade, 2003). These robust oscillations are easily identified using non-invasive EEG and form the main criterion for sleep stage definition across mammalian species (Iber et al., 2007). Both oscillations are implicated in memory consolidation as we review below. While EEG represents summed activity across large cortical territories (Nunez, 1995), we will focus here on accumulating evidence that characterizes slow waves and spindles as local phenomena.

Neocortical slow waves reflect slow (1–4 Hz) alternations of cellular active (up-) and inactive (down-) states of neuronal activity (Steriade et al., 2001; Nir et al., 2011). Although not perfectly coherent, these oscillations represent the most synchronous event in the healthy brain, and traveling waves across large cortical territories may mediate diverse sleep functions including downregulation of synaptic strengths (Vyazovskiy et al., 2008; Norimoto et al., 2018), maintenance of cellular homeostasis (Tononi and Cirelli, 2014), and mediation of memory consolidation and synaptic plasticity (Diekelmann and Born, 2010).

Slow waves are thought to provide a temporal frame for a dialogue between the neocortex and subcortical structures, which is necessary for redistributing memories for long-term storage (Sirota et al., 2003; Sirota and Buzsáki, 2005; Marshall and Born, 2007): On a global scale, a strong increase in EEG coherence is observed during NREM sleep following learning in humans (Mölle et al., 2004, 2009). On a local scale, changes in sleep oscillations occur in specific cortical regions that were involved in encoding, both in rodents (Vyazovskiy et al., 2000; Hanlon et al., 2009) and in humans (Huber et al., 2004, 2006; Mölle et al., 2009). Although very commonly regarded as a global event occurring near-simultaneously across the cortex, cortical up-states are typically ignited locally in prefrontal cortex and spread to other cortical areas over tens to a few 100 ms (Massimini et al., 2004). Neural recordings in rodents were able to pinpoint the ignition source to layer 5 cells of cortex (Luczak et al., 2007; Chauvette et al., 2010; Beltramo et al., 2013). Intracranial recordings from epilepsy patients reveal that most slow waves, and the underlying active and inactive neuronal states, occur locally (Nir et al., 2011). This observation goes beyond potential confounds of epilepsy, since it is readily observed also in rodents and in cats (Chauvette et al., 2011; Vyazovskiy et al., 2011). Especially during late sleep, circumscribed slow waves are also detected via EEG recordings (Siclari et al., 2014; Bernardi et al., 2018).

Sleep spindles are classically defined as waxing-and-waning 10–16 Hz oscillations lasting 0.5–2 s (Gibbs and Gibbs, 1950). Sleep spindles are implicated in plasticity and trigger synaptic long-term potentiation via calcium transients that are believed to prime cortical networks for the long-term storage of memory representations (Timofeev et al., 2002; Rosanova and Ulrich, 2005; Ulrich, 2016; Niethard et al., 2018). On a global scale, increased spindle activity is observed during NREM sleep following learning of both declarative tasks and procedural motor skills (Gais et al., 2002; Eschenko et al., 2006; Fogel and Smith, 2006; Morin et al., 2008; Mölle et al., 2009). On a local scale, regional spindle activity correlates with offline improvement in consolidation of motor memories (Nishida and Walker, 2007). Importantly, despite the fact that spindles engage thalamo-cortical “loops,” they are also mostly a local phenomenon occurring in select circuits at a time (Rasch and Born, 2013). Even when observed near-simultaneously across regions, their precise timings varies across cortical locations (Nir et al., 2011; Muller et al., 2016). Accordingly, learning different types of memories changes the properties of spindles in different topographically-restricted regions (Bergmann et al., 2012; Cox et al., 2014).

**Figure 1:**
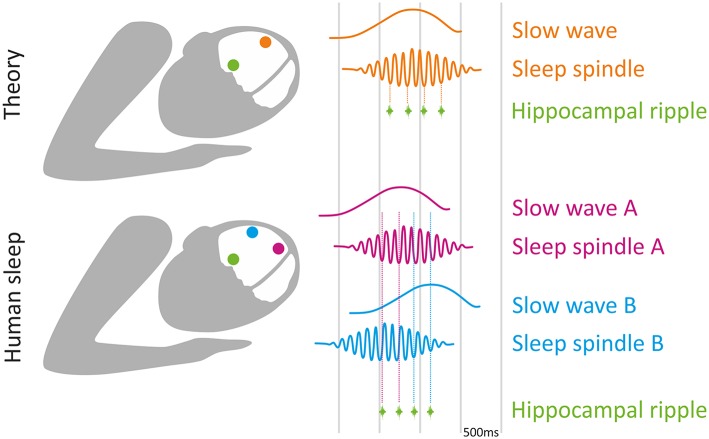
Local sleep oscillations and memory consolidation: theory vs. experimental findings. **(Top)** Theory suggests that the nesting of hippocampal ripples (green) to sleep spindle troughs (orange), which in turn are nested in slow wave up-phase (orange), is critical for memory consolidation during sleep. **(Bottom)** Experimental data indicates that timing of both slow waves and spindles (as well as spindle nesting phase; Andrillon et al., 2011) varies across cortical regions (purple and blue), such that the nesting of each ripple (green) inevitably corresponds to different cortical locations (dashed vertical lines). Thus, each hippocampal ripple occurring at a specific time is associated with hippocampal-cortical coupling in different circuits, likely supporting memory consolidation related to that circuit.

Not only are slow waves and sleep spindles each related to memory consolidation separately, recent evidence suggests that their precise interaction may play a role. For example, many sleep spindles tend to be “nested” in the “up” phase of the slow oscillation as revealed by phase-amplitude coupling (PAC) analysis (Diekelmann and Born, 2010; Staresina et al., 2015). However, slow wave and spindle oscillations behave as traveling waves at a whole-brain scale [for an extensive review see (Muller et al., 2018)], which translates to a delay of up to hundreds of milliseconds between oscillation peaks across different cortical areas. Thus, the temporal relationship between sleep oscillations across cortical regions varies substantially. Locally, within each brain region, the coupling of sleep spindles to slow wave up-states occurs in a topographically restricted fashion (Cox et al., 2014) and local slow waves coordinate spindle activity at virtually every cortical site (Cox et al., 2018). In contrast, coupling between distant brain regions does not necessarily occur regularly. For example, while parietal spindles are coupled to parietal slow waves, they are not necessarily coupled with frontal slow waves (Figure 1). Along this line, the strength of slow wave-spindle coupling differs between global and local slow waves, as well as between cortical locations (Malerba et al., 2019), highlighting the complexity of cross-frequency coupling between sleep oscillations across different brain regions.

## Interregional Coupling Between Hippocampus and Specific Cortical Regions During Sleep, and Its Role in Successful Memory Consolidation

During NREM sleep, hippocampal (HC) activity is concentrated in sharp wave ripple (SWR) events, which correspond to a summed synchronous depolarization of a large fraction of the neurons in the CA1 sub region of the hippocampus (O'keefe and Nadel, 1978; Buzsáki et al., 1983; Buzsáki, 1986). Extensive animal research established a tight link between HC SWRs and memory consolidation in both wakefulness and sleep: SWRs accompany the sleep-associated re-activation of HC neuron ensembles that were active during the preceding awake learning experience (Nadasdy et al., 1999; Eschenko et al., 2008; Peyrache et al., 2009). SWRs occurrence increases in previously potentiated synaptic circuits (Behrens et al., 2005), and may further modulate synaptic strength (Buzsáki et al., 1987; King et al., 1999; Norimoto et al., 2018). Finally, selective manipulation of SWRs through electrical or optogenetic stimulation in HC modulates memory consolidation (Girardeau et al., 2009; Ego-Stengel and Wilson, 2010; Fernandez-Ruiz et al., 2019). Thus, SWRs represent important time epochs for offline HC activity, and their occurrence in NREM sleep carries a privileged role in plasticity and memory consolidation.

In deep layers of medial prefrontal cortex (mPFC), where most of the HC fibers make contacts, pyramidal cells respond phasically to SWRs (Siapas and Wilson, 1998; Mölle et al., 2006; Peyrache et al., 2011). Conversely, the occurrence of SWRs is modulated by neocortical inputs (Isomura et al., 2006), revealing bidirectional interactions between HC and cortex. Multiple studies revealed the fine temporal relationship between SWRs and neocortical sleep oscillations (Sirota et al., 2003; Sirota and Buzsáki, 2005; Staresina et al., 2015; Wang and Ikemoto, 2016), in which SWRs tend to be phase-locked to cortical spindle troughs, which in turn are phase-locked to slow wave up-states. Human studies are typically limited in SWR detection, as non-invasive EEG cannot reliably monitor local high-frequency activities in deep brain structures. Nevertheless, sleep studies in epilepsy patients implanted with intracranial electrodes support the notion that SWRs during sleep preferentially occur at specific times in relation to neocortical slow waves and spindles (Clemens et al., 2007, 2011; Nir et al., 2011; Staresina et al., 2015), extending the temporal tuning finding from rodents to human sleep. Given that spindles are mostly a local phenomenon, and their precise timing varies across cortical locations (Nir et al., 2011; Muller et al., 2016), temporal tuning between one cortical area and HC during a specific spindle does not necessarily imply temporal tuning between other cortical areas to HC at that time (Figure 1).

At present, a gap exists between theory on how hippocampal-cortical coupling supports memory consolidation (usually considering the entire cortex as a uniform entity) and the available experimental evidence highlighting that slow waves, spindles, and SWRs occur at different times in different regions.

## Coupling of Sleep Oscillations in Select Brain Regions and the Consolidation of Specific Memories

A potential way to transcend this discrepancy is to consider that coupling between sleep oscillations may occur, but may involve select circuits at each given time—supporting memory consolidation in specific associated tasks. We illustrate this by considering two recent studies in rodents that causally link the coupling of sleep oscillations across specific regions to the consolidation of specific memories. A recent study (Maingret et al., 2016) established that co-occurrence of HC ripples and medial prefrontal cortex (mPFC) slow waves and spindles correlates with memory consolidation in a spatial learning task. Boosting this coupling by delivering SWR-triggered electrical stimulation to deep cortical layers causally improved memory performance on this hippocampus-dependent task (Maingret et al., 2016). Another study used a different closed-loop stimulation protocol to improve memory performance in a hippocampal dependent task: frontal slow waves triggered optogenetic stimulation of the thalamic reticular nucleus during sleep, resulting in time-locked frontal sleep spindles, and HC SWRs (Latchoumane et al., 2017). Notably, these experiments, as well as studies selectively manipulating SWRs, report changes in coupling between SWRs in a specific hippocampal (HC) sub-field [mostly CA1 (Girardeau et al., 2009; Ego-Stengel and Wilson, 2010; Maingret et al., 2016)], and spindles in specific regions [either mPFC (Siapas and Wilson, 1998) or anterior cingulate cortex (Wang and Ikemoto, 2016)]. Thus, these findings demonstrate that although each SWR may be coupled with slow waves and spindle oscillations in different brain regions (Figure 1), HC-cortical coupling in select circuits may support memory consolidation in specific tasks.

Although the majority of sleep and memory experiments focus on temporal coupling between HC and cortex, several studies also demonstrate the importance of coherence between specific cortical regions. Miyamoto and colleagues demonstrated that coordinating slow wave activity between layer-5 primary somatosensory cortex and secondary motor cortex via synchronous optogenetic stimulation at 2 Hz enhances memory consolidation of a newly learned non-declarative skill. Asynchronous stimulation of these two regions (using opposite phases) reduced performance relative to the no-intervention controls (Miyamoto et al., 2016).

These experiments (Maingret et al., 2016; Miyamoto et al., 2016; Latchoumane et al., 2017) highlight the importance of both temporal and anatomical specificity of interventions designed to boost the coupling between sleep oscillations across two brain regions. Accordingly, a brief delay in stimulation timing was enough to abolish the memory enhancement that is observed when locking stimulation accurately to HC SWRs (Maingret et al., 2016).

## How Can We Improve Causal Interventions in Humans Linking Sleep Oscillations to Learning and Memory?

Over the last decade, several studies have gone beyond demonstrating the existence of correlation between sleep oscillations (slow waves, spindles) and subsequent memory recall (e.g., Gais et al., 2002; Huber et al., 2004, 2006; Mölle et al., 2009; Fogel and Smith, 2011; Van Der Helm et al., 2011; Tamminen et al., 2013), to interventions that link an experimentally-induced increase in the amplitude of a sleep oscillation to human learning (Marshall et al., 2006; Ngo et al., 2013; Ladenbauer et al., 2017, but also see Bueno-Lopez et al., 2019). A recent study demonstrated that causal interventions affecting memory consolidation may also be applied locally. Unilateral olfactory stimulation induced “local targeted memory reactivation” and elicited both behavioral and EEG effects that were largely lateralized to one hemisphere (see preprint at - Bar et al., 2019). Such lateralization seems more difficult to demonstrate in the auditory modality (Simor et al., 2018), possibly because cortical auditory processing is less lateralized compared to vision and olfaction (Schnupp et al., 2011).

One line of causal interventions during sleep employs a temporally tuned approach, to perform “closed-loop” stimulation, phase-locked to endogenous sleep oscillations. For example, auditory stimulation in phase with slow wave up-states (as measured with scalp EEG) enhances slow wave activity and slow wave-spindle coupling, and improves the consolidation of declarative memory (Ngo et al., 2013; Lafon et al., 2017; Ketz et al., 2018; Goldi et al., 2019). Given that the timing of sleep oscillations differs across cortical regions, choosing a specific EEG channel to trigger stimulation, phase-locks the intervention to the timing of a specific cortical region. An elegant human study that took this into consideration shows degradation of learning efficiency following focal perturbation of slow wave activity over the motor cortex (Fattinger et al., 2017). Importantly, the perturbation was ineffective when targeting temporo-parietal cortex slow waves (Fattinger et al., 2017). Such an experimental approach draws our attention to the role of *local* sleep oscillations in specific cortical areas for consolidation of different types of memory tasks. The exact timing of intervention is critical for enhancing memory consolidation, and changing the stimulation phase may abolish memory effects completely (Ngo et al., 2013; Goldi et al., 2019).

Though impossible to directly compare, memory enhancement in humans appears to be modest and less pronounced compared to memory enhancement following interventions manipulating spindles and SWRs in rodents (Maingret et al., 2016; Latchoumane et al., 2017). We suggest that the precise timing of the intervention is critical for memory enhancement and may constitute an obstacle we need to overcome to obtain larger effects in human subjects. At present, human interventions typically rely on scalp EEG summating neuronal activity across wide regions, whereas animal studies track activity of specific neural populations in deep brain areas.

When studying coherence of EEG sleep oscillations between different cortical sites in humans, an important consideration is the tight and often underappreciated relation between (i) the amplitude of a sleep oscillation (e.g., slow wave or sleep spindle) as recorded with scalp EEG or intracranially, and (ii) its coherent occurrence across neuronal ensembles. Put simply, high-amplitude oscillations often reflect high synchronization between neuronal populations. Indeed we have shown, based on local iEEG recordings, that the amplitude of each slow wave recorded on the scalp is tightly correlated with the number of distant brain regions where this wave occurs near-simultaneously, such that high-amplitude slow waves are global (Nir et al., 2011). In the case of sleep spindles, high-amplitude events in scalp EEG likely reflect a precise coordination among neurons in cortex, thalamus, and reticular thalamic nucleus (Nunez, 1995). This means that many findings that link EEG slow wave or spindle amplitude/power in a given region to learning and memory may in fact imply stronger coherence within relevant neuronal circuits. Notwithstanding this, other factors also influence the amplitude of EEG sleep oscillations, as asynchronous local generators can also produce an unexpectedly large scalp signal (Von Ellenrieder et al., 2016). Further research is needed in order to separate the contribution of high oscillatory power vs. high coherence between specific areas to memory consolidation.

## Future Outlooks

Technological advances should allow accurate mapping of the roles of specific spatially-circumscribed cortical sleep events in the consolidation of long term memory in humans, and separate them from other functions carried out by events that travel and encompass the whole cortex. We expect that maturation of novel electrophysiology tools will improve both spatial and temporal resolutions of monitoring human brain activity in real-time (Khodagholy et al., 2017; Liu et al., 2018), thereby allowing accurate experimental interventions in humans and improving their electrophysiological and cognitive effects. For such advances to make an impact on basic scientific understanding and create genuine clinical utility, it is imperative that theory is fine-tuned according to the available data, and that we go beyond considering the sleeping brain as a uniform coherent entity.

## Author Contributions

All authors listed have made a substantial, direct and intellectual contribution to the work, and approved it for publication.

### Conflict of Interest Statement

The authors declare that the research was conducted in the absence of any commercial or financial relationships that could be construed as a potential conflict of interest.
